# Special Issue “Recent Process Design and Development Strategies for Dental Materials”

**DOI:** 10.3390/ijms25105057

**Published:** 2024-05-07

**Authors:** Mary Anne S. Melo

**Affiliations:** Department of Comprehensive Dentistry, School of Dentistry, University of Maryland, 650 W. Baltimore St., Baltimore, MD 21201, USA; mmelo@umaryland.edu

The field of dental materials is rapidly evolving, and this Special Issue of the *International Journal of Molecular Sciences* offers a comprehensive examination of the latest advancements in process design and development strategies [1]. The articles compiled herein provide a diverse yet focused exploration of the novel materials, techniques, and methodologies shaping the future of restorative treatments and new restorative material solutions.

The research presented in this Special Issue highlights significant innovations in bioactive materials, such as 3D-printed bioglass and advanced resin composites [2]. These materials are notable for their improved biocompatibility and enhanced performance characteristics in dental restorations, offering superior mechanical properties and clinical outcomes [3].

Furthermore, work on nanotechnology and its integration into dental materials is a recurring theme throughout this issue [4]. Innovative approaches to nanoparticle incorporation are shown to significantly advance the functional properties of materials, aiding in everything from antibacterial capabilities to the remineralization of tooth enamel.

Another pivotal aspect covered is the environmental and human health considerations related to dental material processing [5]. Investigations into the cytotoxicity of resin-based materials and environmental assessments provide critical insights into the safety and sustainability of dental materials [6].

This issue also touches upon the technological advancements in manufacturing processes, such as computer-aided design and computer-aided manufacturing (CAD/CAM) and selective laser melting (SLM) techniques, which are integral to the customization and precision of dental restorations [7]. These technologies have great potential to impact treatment outcomes and patient-specific solutions positively.

In conclusion, this Special Issue presents cutting-edge research and sets the stage for future developments in dental materials. It is clear that interdisciplinary approaches, combining materials science, engineering, and clinical expertise, are essential to overcoming current challenges and unlocking new possibilities in restorative dentistry. The ongoing collaboration between researchers, clinicians, and industry stakeholders is crucial to the continued innovation and implementation of these advanced materials and technologies.
